# Discovery of a novel binding pocket in PPARγ for partial agonists: structure-based virtual screening identifies ginsenoside Rg5 as a partial agonist promoting beige adipogenesis

**DOI:** 10.3389/fchem.2025.1579445

**Published:** 2025-05-08

**Authors:** Zhen Wang, Kexin Shui, Zehui Zhang, Yihan Chen, Nanfei Yang, Shiliang Ji, Pingping Shen, Qiang Tian

**Affiliations:** ^1^ State Key Laboratory of Pharmaceutical Biotechnology, Department of Urology, The Affiliated Nanjing Drum Tower Hospital, The Affiliated Hospital of Nanjing University Medical School, School of Life Sciences, Nanjing University, Nanjing, China; ^2^ Suzhou Research Center of Medical School, Suzhou Hospital, Affiliated Hospital of Medical School, Nanjing University, Suzhou, China; ^3^ Department of Colorectal Surgery, The First Affiliated Hospital of Wenzhou Medical University, Wenzhou, China

**Keywords:** PPARγ, binding pocket, natural product, virtual screening, beige cells

## Abstract

Peroxisome proliferator-activated receptor gamma (PPARγ) is a key target for metabolic disorders that contribute to obesity and type 2 diabetes mellitus (T2DM). However, full agonists such as thiazolidinediones (TZDs) have limitations in terms of side effects. Selective PPARγ modulators (SPPARγMs) that target alternative binding pockets offer the potential for safer partial agonists. Here, we employed six computational algorithms (Fpocket, DeepSite, CavityPlus, DoGSiteScorer, CASTpFold, POCASA) to identify a novel allosteric pocket (pocket 6–5) in the PPARγ ligand-binding domain (LBD), localized at the helix 3 (H3), helix 2 (H2), helix 2'(H2′), and β-sheet interface. A virtual screening of 4,097 natural compounds from traditional Chinese medicine (TCM) libraries was conducted, which led to the identification of ginsenoside Rg5 (TWSZ-5) as a top hit. Molecular docking and molecular dynamics (MD) dynamics revealed TWSZ-5 stabilizes pocket 6–5 through hydrogen bonds with Ser342, Gln345, Lys261, and Lys263. TWSZ-5 promoted beige adipocyte differentiation in adipose-derived stem cells (ADSCs) *in vitro*, upregulating Ucp1, Prdm16, Cpt1α, and Pgc1α. The present study identifies TWSZ-5 as a novel SPPARγM that utilizes an allosteric binding pocket to enhance thermogenesis while mitigating adverse effects. These findings emphasize the potential of TCM derivatives and structure-based screening strategies to develop safer antidiabetic therapies with precision pharmacology.

## 1 Introduction

Peroxisome proliferator-activated receptor gamma (PPARγ), a ligand-activated transcription factor belonging to the nuclear receptor superfamily, plays a pivotal role in adipocyte differentiation, glucose homeostasis, and lipid metabolism (Gross et al., 2017; Khan et al., 2019; Montaigne et al., 2021). As a master regulator of metabolic processes, PPARγ has been extensively targeted for the treatment of T2DM and metabolic syndrome (Polyzos et al., 2017; Philipson, 2020). The canonical activation mechanism involves ligand binding to the orthosteric pocket within the LBD, inducing conformational changes that recruit coactivators such as *Prdm16* and *Pgc1α* (Piccinin et al., 2019; Shen et al., 2022). The Y-shaped LBD, comprising 13 α-helix and 4 β-sheets, has been observed to exhibit a propensity for stabilization of helix 12 (H12) in an active conformation by full agonists such as thiazolidinediones (TZDs) (Sauer, 2015; Miyachi, 2021). However, the clinical utility of TZDs, such as rosiglitazone (RSG) and pioglitazone, have been severely limited by adverse effects including weight gain, fluid retention, and cardiovascular risks (Bansal et al., 2020; Hall et al., 2020; Kounatidis et al., 2025). It is attributed to their full agonism leading to indiscriminate coactivator recruitment.

Recent advances in PPARγ pharmacology highlight the therapeutic potential of partial agonists that selectively modulate receptor activity. This concept is referred to as selective PPARγ modulation (SPPARγM), which act on PPARγ through an H12-independent mechanism (Bruning et al., 2007; Huang et al., 2021; Miyachi, 2021). The SPPARγMs have been shown to induce submaximal activation function 2 (AF-2) stabilization while preserving insulin sensitization, thereby mitigating adverse effects through differential cofactor binding (Jiang et al., 2020). Notably, structural plasticity of the PPARγ LBD allows the existence of alternative binding sites beyond the orthosteric pocket. For instance, the recently identified a novel pocket adjacent to H3, H2’ and the β-sheet region has emerged as a promising target for allosteric modulators (Tian et al., 2024). Exploiting such novel pockets could enable the development of ligands with unique pharmacodynamic profiles by altering the spatial occupancy and dynamic interactions within the LBD.

Despite these insights, current PPARγ drug discovery faces two critical challenges: limited structural diversity among existing partial agonists, most of which still occupy the canonical Y-shaped pocket with suboptimal subtype selectivity; Insufficient understanding of how alternative binding modes translate to functional selectivity (Ma et al., 2021). The PPARγ partial agonists, including CS-7017, INT-131, lobeglitazone, and TAK-559, commonly adopt a tripartite structural motif consisting of a “carboxylic acid head group-phenyl core-lipophilic tail” (Supplementary Figure S1) (Frkic et al., 2017; Shinozuka et al., 2018; Bae et al., 2021). The carboxylic acid head group forms a hydrogen-bonding network with residues such as Ser289, His323, and Tyr473 in the Y-pocket (Sauer, 2015). The phenyl core is anchored in the hydrophobic cavity through π-π stacking. Meanwhile the flexible lipophilic tail chain extends into the H3-H5 region without breaching the overall conformational constraints of the Y-pocket (Kim et al., 2020). This design paradigm has resulted in over 80% of candidate molecules still being based on thiazolidinedione or carboxylic acid scaffolds (Garcia-Vallvé et al., 2015), resulting in significant chemical space homogeneity. However, this structural characteristic leads to potential risks associated with PPARγ activation mechanisms (Mandal et al., 2022). Furthermore, PPARγ exhibits up to 60% sequence homology with PPARα/δ in the LBD, particularly with key residues such as Tyr473, His323 of the Y-pocket being fully conserved across subtypes. This conservation may lead to cross-activation risks. For example, fenofibrate, a pan PPARα/γ partial agonist,has been associated with hepatotoxicity (Zhang et al., 2022). Bezafibrate, a triple PPARα/δ/γ partial agonist, exhibits mitigated gastrointestinal toxicity by preferentially activating intestinal PPARδ (Nasser et al., 2023). Therefore, the discovery of novel binding pockets within LBD holds critical significance for the identification of innovative scaffold compounds and the advancement of drug discovery. Virtual screening (VS) strategies leveraging high-resolution crystal structures and molecular dynamics (MD) simulations offer a powerful approach to address these gaps (Carlsson and Luttens, 2024; Zhao, 2024). By focusing on novel binding pockets, computational methods can prioritize compounds that engage unique interaction networks, potentially unlocking “druggable” chemical space inaccessible to traditional screening libraries.

This study aims to identify novel PPARγ partial agonists through structure-based virtual screening targeting a newly discovered binding pocket in the LBD. Six widely used algorithms were employed to detect the novel binding pocket of PPARγ LBD. Our findings indicate that pocket 6–5 is the most susceptible to partial agonists interaction, with its location in the H3, H2, H2’, and β-sheet. Using molecular docking and binding free energy calculations, we systematically evaluated compound libraries to discover ligands that stabilize a distinct receptor conformation associated with partial agonism. Our results showed that TWSZ-5 was able to bind in the pocket 6-5 and partially activate PPARγ. Furthermore, it was observed to be highly active in terms of promoting the differentiation of beige adipocytes. Our work not only expands the structural repertoire of PPARγ modulators but also provides mechanistic insights into ligand-induced cofactor selectivity, offering a blueprint for developing safer antidiabetic agents with precision pharmacology.

## 2 Materials and method

### 2.1 Chemicals, reagents and plasmids

Commercial chemicals and reagents, including HPLC-grade ginsenoside Rg5 (HY-N0908, MCE, United States) with a purity level of 99.87%, rosiglitazone (RSG) (HY-17386, MCE, United States), a BODIPY 493/503 Staining Kit (C2053S, Beyotime, China), a dual-luciferase reporter assay kit (E1910, Promega, United States), and a AceQ qPCR SYBR Green Master Mix kit (Q111-02, Vazyme, China), were purchased. PPRE-luc (E4121) and pRL (E2261) plasmids were purchased from Promega. The cDNA sequence of PPARγ was cloned into the pLenti6/v5 lentiviral expression vector using the homologous recombination method.

### 2.2 The prediction of binding pockets

The potential pockets of PPARγ (PDB: 9F7W) were predicted using the Fpocket (https://fpocket.sourceforge.net/), DoGSiteScorer (https://proteins.plus/), POCASA (https://g6altair.sci.hokudai.ac.jp/g6/service/pocasa/), CavityPlus (http://www.pkumdl.cn:8000/cavityplus/index.php), DeepSite (https://open.playmolecule.org/tools/deepsite), CASTpFold (https://cfold.bme.uic.edu/castpfold/). Default parameters were used for all calculations. The potential binding pockets in PPARγ LBD were visualized using PyMoL 3.0 (Schrodinger, United States).

### 2.3 In-house library preparation

This chemical library is based on our previous research, which contains 4,097 natural compounds derived from TCMs (Tian et al., 2024). All molecular structures were entered into Maostro 11.5 (Schrodinger, United States) for ligand preparation under the OPLS3 force field.

### 2.4 Molecular docking and binding energy calculation

Molecular docking studies were performed using the Glide module (version 11.5, Schrödinger 2018-1) within the Schrödinger Suite (Schrodinger, United States) to identify potential PPARγ partial agonists targeting the pocket 6–5 in PPARγ LBD. The PPARγ LBD crystal structure (PDB: 9F7W) was prepared using the Protein Preparation Wizard, including hydrogen bond optimization, restrained minimization (OPLS3 force field), and removal of crystallographic water molecules. A receptor grid (15 Å × 15 Å × 15 Å) centered on pocket 6-5 was generated using default parameters (van der Waals scaling factor = 0.8; charge cutoff = ± 0.25). Virtual screening was conducted in flowing three sequential precision modes. High-throughput virtual screening (HTVS) to rapidly filter 4,097 natural compounds from our TCM library; Standard precision (SP) refinement of top HTVS hits (GlideScore ≤ −5.0); Extra precision (XP) re-docking to eliminate false positives and optimize pose rankings (Figure 3A). Post-docking prioritization integrated dual criteria. Calculated partition coefficients (Log P) were determined via the QikProp module, retaining compounds with Log P < 5.0 to ensure favorable pharmacokinetic profiles; Molecular mchanics generalized born surface area (MM-GBSA) calculations using the Prime module were performed on XP-docked poses for binding free energy Fassessment. Final candidates were selected based on consensus scores (GlideScore + ΔG bind) and visual inspection of hydrogen-bonding networks with key residues using PyMOL 3.0.

### 2.5 Molecular dynamics (MD) simulations

Molecular dynamics (MD) simulations were conducted in GROMACS 2022 to assess the stability of the PPARγ-TWSZ-5 complex. The protein and ligand were parameterized using the CHARMM36 force field and GAFF2, respectively. The complex was immersed in a cubic TIP3P water box with 1.2 nm periodic boundary conditions. Then, 0.15 M NaCl was added using the genion tool for neutralization. Electrostatic and van der Waals interactions were calculated using the Particle Mesh Ewald (PME) method and a 1.0 nm cutoff. After energy minimization (5,000 steps; force convergence <1,000 kJ·mol^-1^·nm^-1^), the system underwent sequential equilibration: 100 ps NVT (310 K) and 100 ps NPT (1 bar). Production simulations ran for 100 ns under controlled temperature (V-rescale thermostat) and pressure (Parrinello-Rahman barostat). Trajectory frames were saved every 10 ps for subsequent analysis. Key metrics included: Structural stability: Backbone root-mean-square deviation (RMSD) calculated via the Kabsch algorithm; Hydrogen bond occupancy: Defined by donor-acceptor distance ≤3.5 Å and angle ≤30°; Radius of Gyration (Rg): Computed using gmx gyrate to evaluate global compactness by measuring the root-mean-square distance of backbone atoms from the system’s centroid; Solvent-Accessible Surface Area (SASA): Analyzed via gmx sasa with a 0.14 nm solvent probe radius to quantify solvent exposure of the complex, including per-residue contributions and total SASA over time.

### 2.6 PPARγ transactivation assays

The dual-luciferase reporter assay was utilized to assess PPARγ transcriptional activation in our established experimental system (Yang et al., 2023). HEK293T cells were seeded at a density of 5 × 10^4^ cells/well in 24-well plates and transfected with PPARγ expression plasmid, a PPRE-driven firefly luciferase reporter (PPRE-luc), and a constitutive Renilla luciferase control plasmid (pRL) using Lipofectamine 2000 (11,668,030, Invitrogen, United States). Twenty-four hours post-transfection, cell lysates were subsequently collected. Firefly luciferase activity was measured and normalized to Renilla luciferase activity to account for transfection efficiency.

### 2.7 Microscale thermophoresis (MST)

The binding affinity of TWSZ-5 with PPARγ-LBD was quantified via MST following established protocols (Khavrutskii et al., 2013). Briefly, HEK293 cells expressing GFP-tagged PPARγ were lysed in RIPA buffer (P0013B, Beyotime, China) supplemented with protease inhibitors (HY-K0010, MCE, United States). Total protein concentration was determined using a bicinchoninic acid (BCA) assay. Subsequently, the lysate was diluted in MST buffer containing 50 mM Tris-HCl (pH 7.4), 250 mM NaCl, 10 mM MgCl_2_, 0.05% Tween-20, and 5% bovine serum albumin. Protein-ligand mixtures were introduced into Monolith NT.115 capillaries (NanoTemper, Germany), and thermophoretic mobility was analyzed with MO Control v1.6 software.

### 2.8 The assay of CCK-8

ADSCs were counted and approximately 4,000 cells per well were seeded in a 96-well plate. After 24 h of incubation at 37°C in a humidified 5% CO_2_ atmosphere, the medium was replaced with varying concentrations of TWSZ-5, diluted in the corresponding culture medium. Three replicates were performed for each concentration, and cells were incubated for an additional 24 h. Following incubation, 10 μL of CCK-8 reagent (C0037, Beyotime, China) was added, and absorbance at 450 nm was measured using a multifunction microplate reader (AMR-100, Allsheng, China) after 3 h at 37°C. Cell viability was calculated as the percentage of each concentration relative to the control.

### 2.9 Primary adipocyte differentiation

Primary preadipocytes were cultured in DMEM/F12 medium (L310KJ, Basalmedia, China) supplemented with 10% FBS (10099141C, SenBeiJia, Thermo, United States) at 1 × 10^5^ cells/mL, maintained at 37°C in 5% CO_2_. Differentiation was initiated at 100% confluence using induction medium containing: DMEM/F12 base, 10% FBS, 1 mM IBMX (HY-12318, MCE, United States), 1 µM RSG, 10 μg/mL insulin (HY-P0035, MCE, United States), 5 µM dexamethasone (HY-14648, MCE, United States), 1 nM triiodothyronine (HY-A0070A, MCE, United States), 125 nM indomethacin (HY-14397, MCE, United States), and 1% penicillin/streptomycin (ST488S, Beyotime, China). After 3 days, the medium was replaced with maintenance medium (DMEM/F12 with 10 μg/mL insulin and 10% FBS), refreshed every 2–3 days. Differentiated adipocytes were analyzed between Days 7–14 post-induction, with lipid droplet accumulation confirmed by light microscopy.

### 2.10 BODIPY staining

BODIPY dye was used to stain lipid droplets in adipocytes. Cells were fixed with 4% paraformaldehyde (G1101-500, Servicebio, China) for 20 min at room temperature. The cells were then washed with PBS twice, and stained with a freshly prepared solution containing BODIPY 493/503 (1:1000) and DAPI (1:1000) (C1005, Beyotime, China) for 20 min at room temperature. After washing, lipid droplets (green, 488/503 nm) and nuclei (blue, 350/461 nm) were visualized using fluorescence microscopy (BX53, Olympus, Japan).

### 2.11 Cell culture

HEK293T cells obtained from the Stem Cell Bank (Chinese Academy of Sciences) were cultured in DMEM supplemented with 10% FBS, 1× penicillin-streptomycin, and 2 mM glutamine. For primary adipocyte isolation, minced adipose tissue from C57BL/6J mice was digested with 125 U/mg collagenase II (40508ES76, Beyotime, China) at 37°C for 1 h, and after filtration (100 µm), the mixture was centrifuged (300 g, 10 min, 4°C) to isolate preadipocytes. Adipose-derived stem cells (ADSCs) were maintained in DMEM/F12 with 15% FBS and 1× penicillin-streptomycin-glutamine.

### 2.12 Quantitative real-time PCR (q-PCR)

Total RNA was isolated from cells using TRIzol reagent (R401-01, Vazyme, China). Reverse transcription was performed with HiScript III RT SuperMix kit (R323-01, Vazyme, China) according to the manufacturer’s instructions. Quantitative RT-PCR assays were performed on a real-time PCR system (CFX opus96, Bio-Rad, United States) using qPCR SYBR Green Master Mix.

### 2.13 Statistical analysis

All results are expressed as mean ± SD. Statistical analysis was performed with two-tailed Student’s t-test or one-way ANOVA followed by Dunnett’s test or Tukey’s test using Prism 9.0 (GraphPad, United States). P ≤ 0.05, p ≤ 0.01, and p ≤ 0.001 were considered statistically significant and are indicated by *, **, and ***, respectively.

## 3 Results

### 3.1 Identification of small molecule-binding pockets in PPARγ LBD

**FIGURE 1:**
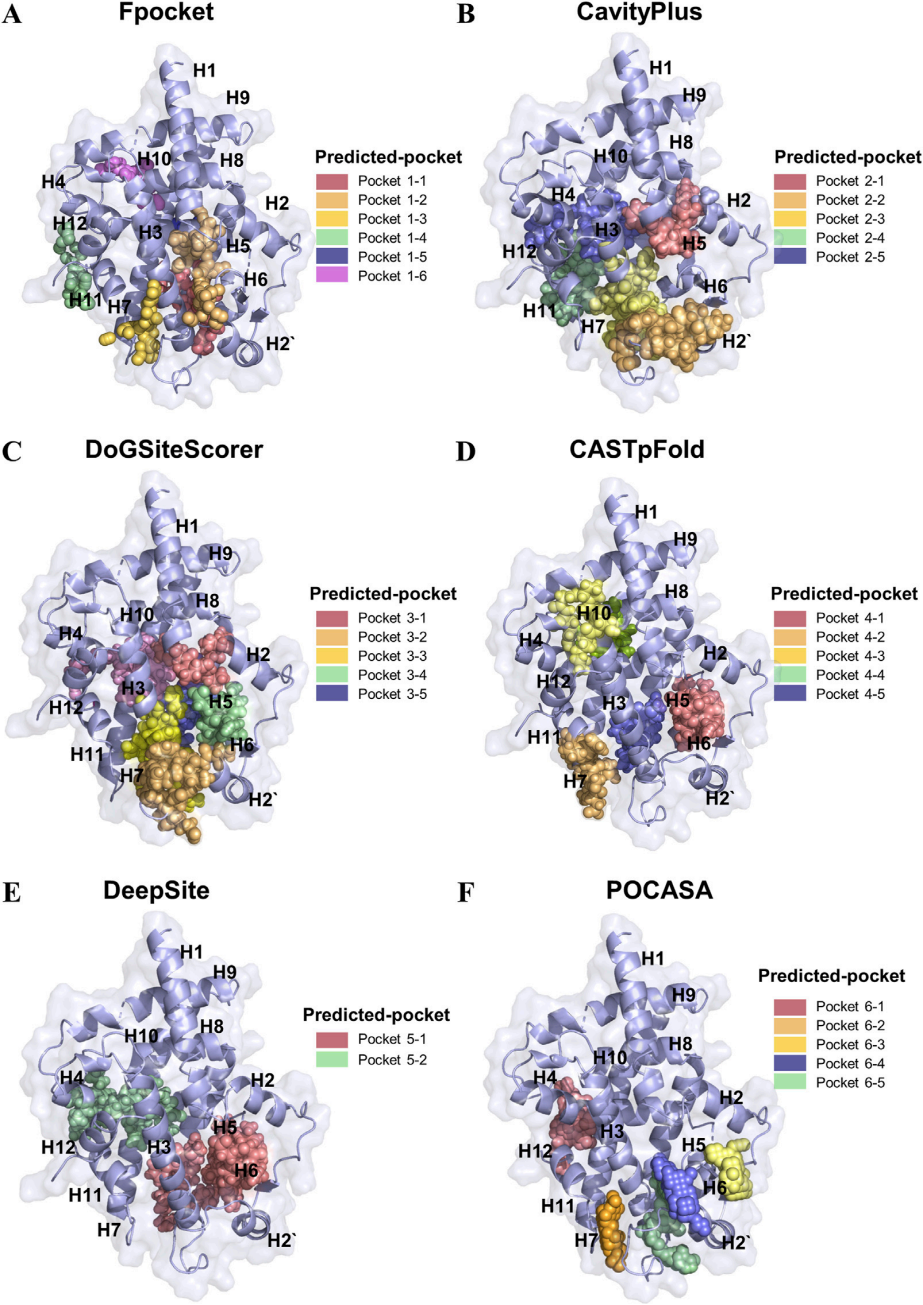
Identification of the binding pockets in PPARγ LBD using different tools. **(A)** Fpocket prediction of six high-rank possible binding pockets in PPARγ LBD. **(B)** CavityPlus prediction of five high-rank possible binding pockets in PPARγ LBD. **(C)** DoGSiteScorert prediction of five high-rank possible binding pockets in PPARγ LBD **(D)** CASTpFold prediction of five high-rank possible binding pockets in PPARγ LBD. **(E)** DeepSite prediction of two high-rank possible binding pockets in PPARγ LBD. **(F)** POCASA prediction of five high-rank possible binding pockets in PPARγ LBD.

To systematically identify potential ligand-binding pockets within the LBD of PPARγ, we employed six complementary computational tools: Fpocket, Deepsite, CavityPlus, DoGSiteScorer, CASTpFold, and POCASA (Figure 1). These algorithms were utilized to predict and cross-validate pocket characteristics, including volume, depth, surface accessibility, and physicochemical properties. The consensus predictions identified a dominant binding pocket localized near the H3-H12 interface, a region critical for co-activator binding and ligand-induced conformational changes. Structural alignment revealed overlapping predictions across all tools for this primary pocket. Notably, Fpocket (Figure 1A) and CavityPlus (Figure 1B) demonstrated superior spatial agreement in delineating the pocket boundaries, particularly around residues H3, H4, and H12, which are functionally linked to ligand recognition. Secondary pockets near the β-sheet region (H2-H6 loop) and the H7-H11 interface were inconsistently predicted, with tool-specific variations in volume and accessibility metrics (Figures 1C–F). Consensus analysis revealed six high-probability binding pockets in the LBD region, with Fpocket demonstrating superior spatial resolution in delineating pocket boundaries. The top-ranked pocket, located near the H3-β sheet loop interface (pocket 1–1, pocket 2–2, pocket 3–3, pocket 4–5, pocket 5–1, and pocket 6–5), exhibited conserved residues critical for co-activator recruitment and ligand interactions, as supported by overlapping predictions from all six methods. Geometric clustering analysis prioritized the H3-H12 pocket for molecular docking due to its evolutionary conservation (>80% residue identity across homologs) and congruence with crystallographic ligand-binding sites. Divergent predictions from individual tools were resolved through consensus scoring, minimizing false-positive identifications. This integrative approach establishes a robust framework for targeting PPARγ LBD in structure-based drug design.

### 3.2 The pocket 6–5 exhibited optimal druggability

**FIGURE 2:**
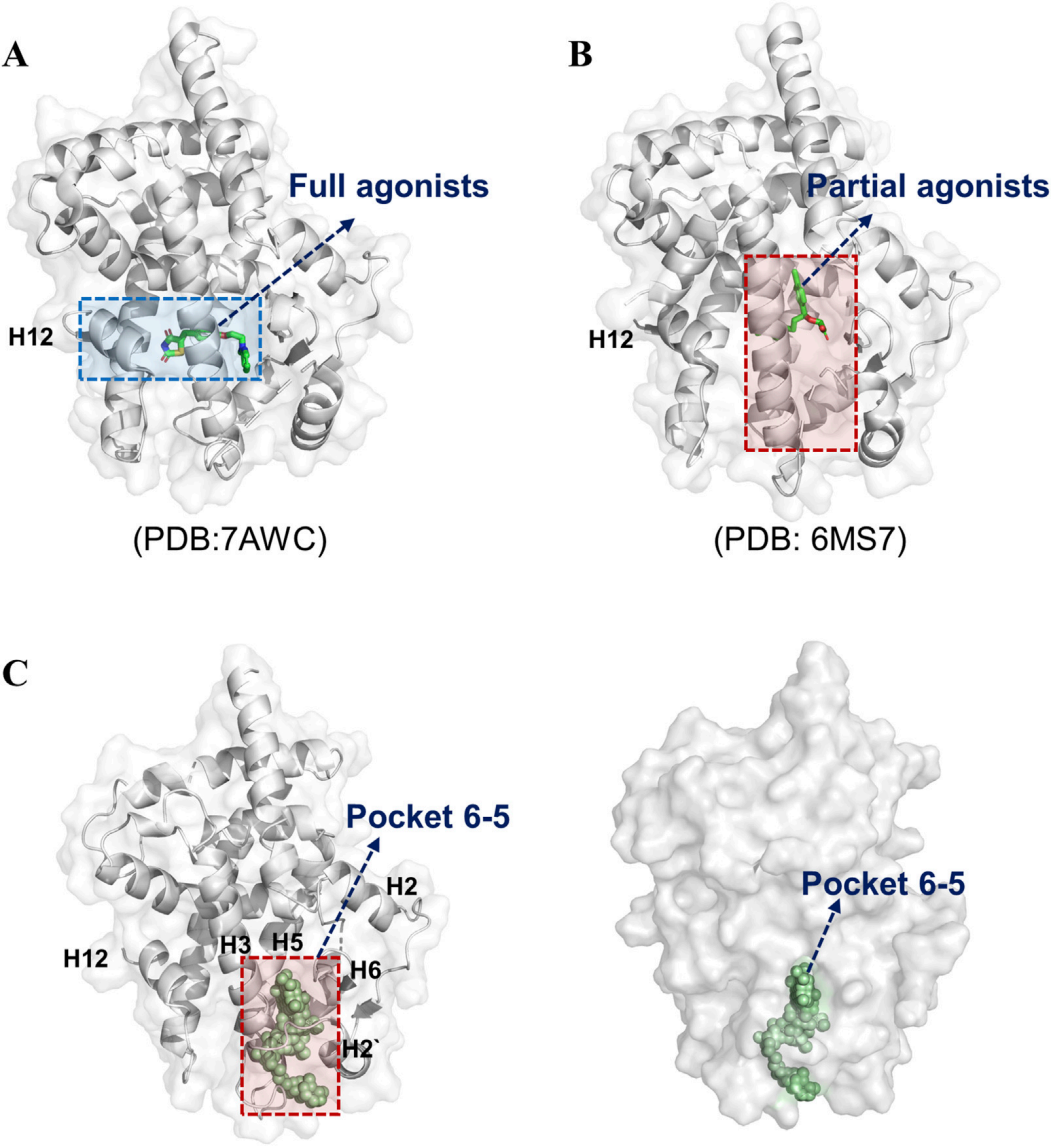
Pocket 6–5 was located in the H3-H2-H2′ cavity. **(A)** The crystal structure of RSG-PPARγ LBD complex. **(B)** The crystal structure of VSP77-PPARγ LBD complex. **(C)** The detail location information of pocket 6–5.

The LBD of PPARγ exhibits distinct interaction patterns between full and partial agonists, as illustrated in Figure 2. Full agonists, such as rosiglitazone and pioglitazone, stabilize the active conformation by forming direct hydrogen bond contacts with residues on H12, a critical structural element for coactivator recruitment (Figure 2A). In contrast, partial agonists bypass interactions with H12 and instead mediate receptor activation through alternative mechanisms. For instance, VSP-77 adopts an active conformation characterized by tight engagement of H3 and H4 in the LBD (Figure 2B). This interaction stabilizes the binding region independently of H12 positioning, suggesting a unique mode of partial agonism. Notably, the binding pocket of partial agonists involves residues from helices H3, H4, and H11, which may collectively contribute to a distinct conformational rearrangement that supports intermediate transcriptional activity. This unique binding mode suggests a structural basis for partial agonism, wherein stabilization of the AF-2 interface, rather than H12 positioning, drives transcriptional activation. Thus, based on the above results, we choose the pocket 6-5 for further study. Pocket 6-5 was localized at the interface of helix H3-H2-H2′ region, as depicted in Figure 2C. This cavity exhibits a conserved architecture, with a pronounced hydrophobic core flanked by polar residues on the H3 and the H2′ surface. The pocket’s geometry accommodates ligand stabilization through complementary van der Waals interactions with hydrophobic side chains and hydrogen-bonding networks. Notably, the spatial alignment of H3 and H2’ creates a contiguous binding surface that facilitates ligand-induced conformational stabilization of the receptor. This unique arrangement suggests a cooperative mechanism whereby ligand occupancy in the H3-H2-H2′ cavity promotes the repositioning of the AF-2 helix, a critical step for coactivator recruitment and transcriptional activation. The observed structural features underscore the functional significance of pocket 6-5 in mediating PPARγ agonism.

### 3.3 Screening of PPARγ agonists within the in-house chemical library

**FIGURE 3:**
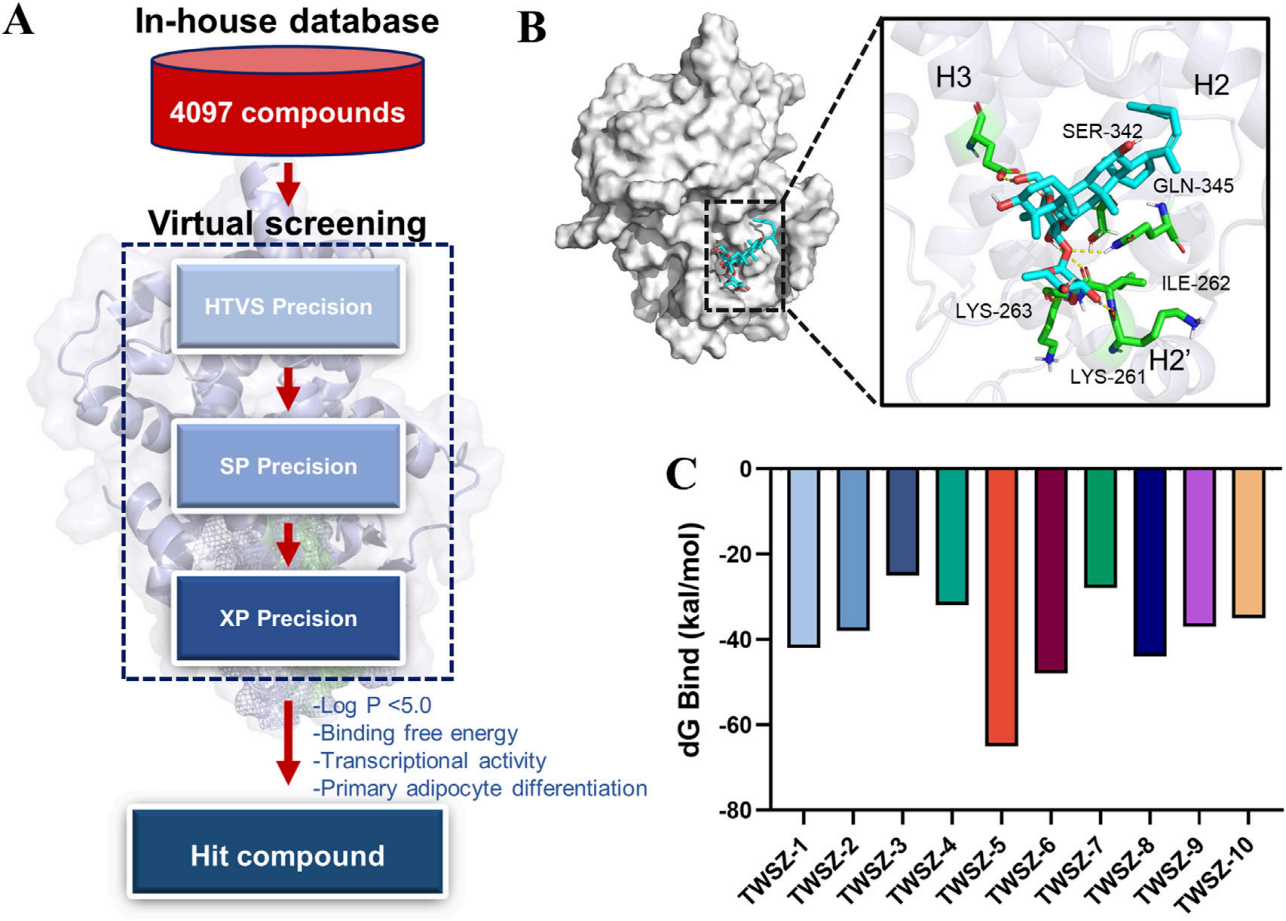
Virtual screening identifies TWSZ-5 binding in pocket 6–5. **(A)** The workflow of structure-based virtual screening based on in-house natural compound library. **(B)** The docking model of TWSZ-5 in the pocket 6–5 **(C)** MM-GBSA analysis of the binding free energies of the top 10 compounds to PPARγ LBD.

Based on the above results, we choose the pocket 6-5 for further virtual screening. Virtual screening of 4,097 natural product compounds from our in-house database was performed using Glide modules (HTVS, SP, and XP) to identify potential PPARγ ligands (Figure 3A). Initial hits were refined by applying multi-tiered criteria, including Log P < 5.0, binding free energy, which culminated in the identification of TWSZ-5 as a top candidate with docking score −8.292 (Table 1). Molecular docking analysis revealed that TWSZ-5 occupies the PPARγ LBD pocket adjacent to helices H3, H2, and H2’, forming hydrogen bonds with Ser342, Gln345, Lys263, Lys261, and Ile262 (Figure 3B). TWSZ-6 formed hydrogen-bonding interactions with Glu295, Glu291, Glu343, Gln345, Met257, Lys261, and Ser245 with PPARγ LBD (Supplementary Figure S2A). TWSZ-8 formed six hydrogen bonds with Glu291, Gln345, Ser342, Lys262, Lys244, and Ile262 (Supplementary Figure S2B). TWSZ-1 and TWSZ-2 could also target PPARγ with the docking scores of 7.923 and 7.852, respectively (Supplementary Figures S2C, D). TWSZ-9 formed hydrogen bonds with Ser342, Gln345, Lys265, Asp243, Glu-291, and Lys263 (Supplementary Figure S2E) with the docking score was 7.416. TWSZ-4, TWSZ-7, and TWSZ-3 had lower affinity to PPARγ with the docking scores were 6.358, 6.559, and 5.872, respectively (Supplementary Figures S2G–I). Binding stability was further validated by MM/GBSA scoring, where TWSZ-5 exhibited the highest binding affinity (−65.879 kcal/mol), significantly outperforming other candidates such as TWSZ-6 (−48.458 kcal/mol) (Figure 3C).

**TABLE 1:** The docking sores of active compounds.

ID	Doccking score	Glide energy(Kcal/mol)
TWSZ-5	−8.292	−65.879
TWSZ-6	−8.065	−48.458
TWSZ-8	−7.971	−44.785
TWSZ-1	−7.923	−42.321
TWSZ-2	−7.852	−38.298
TWSZ-9	−7.416	−37.695
TWSZ-10	−7.079	−35.412
TWSZ-4	−6.358	−32.256
TWSZ-7	−6.229	−28.325
TWSZ-3	−5.872	−25.145

According to the dG Bind value, we choose the TWSZ-5, TWSZ-6, TWSZ-8, and TWSZ-1 compounds for evaluating the bioactivity. As demonstrated in Supplementary Figure S3, these compounds exhibited divergent adipogenic effects. Among them, TWSZ-5 exhibited the strongest adipogenic capacity, followed by TWSZ-6 and TWSZ-8 (Supplementary Figure S3A). This phenomenon is consistent with the expression of beige cell-related marker genes, including Ucp-1, Prdm16, and Pgc1α (Supplementary Figure S3B). Based on its superior docking score, interaction profile, and bioactivity, TWSZ-5 was selected for in-depth biological evaluation.

### 3.4 TWSZ-5 showed a better transcriptional activity of PPARγ

**FIGURE 4:**
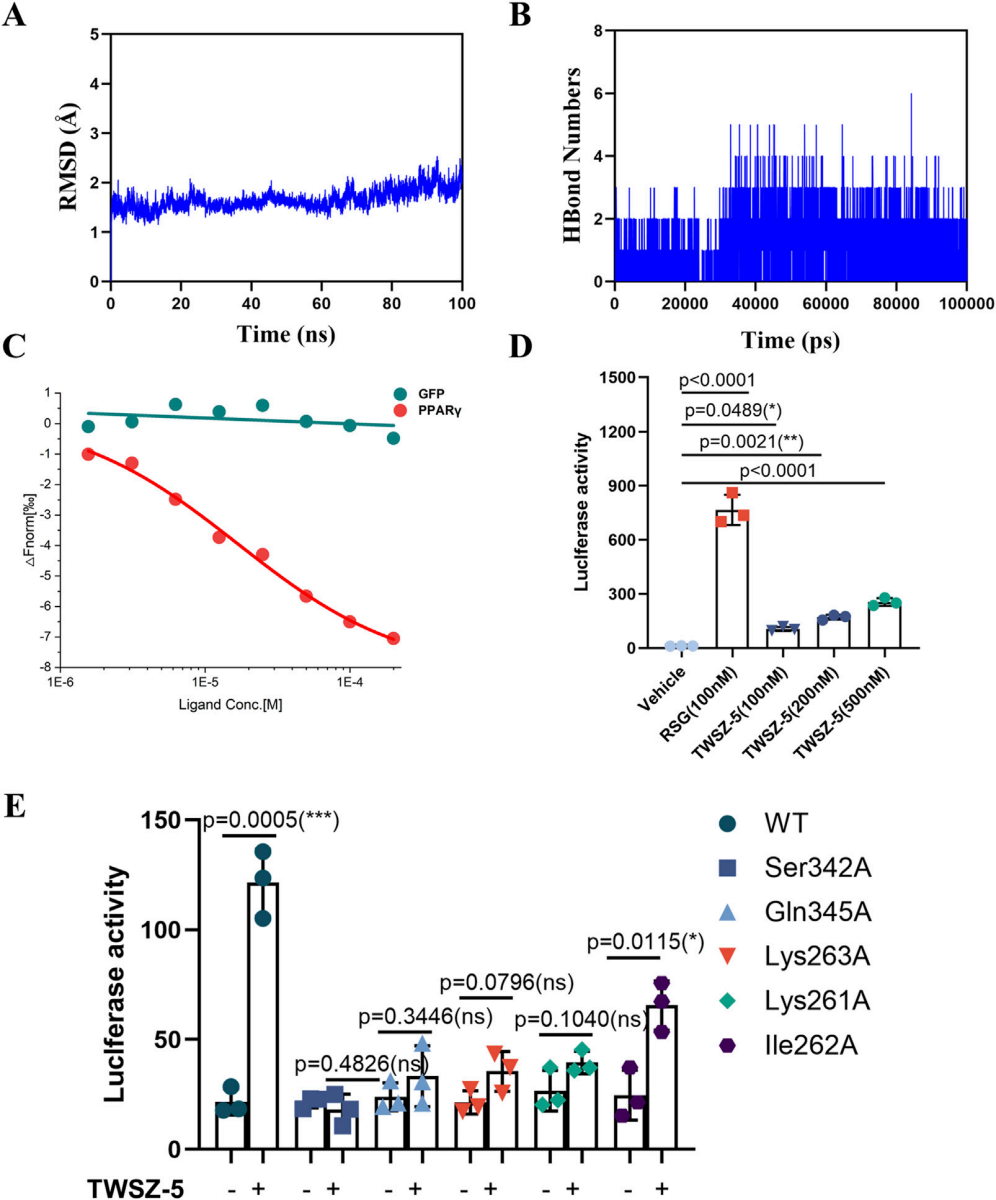
TWSZ-5 could target and active PPARγ. **(A)** The RMSD analysis of TWSZ-5-PPARγ complex. **(B)** The HBond numbers of TWSZ-5- PPARγ complex. **(C)** MST assay tested the interaction of TWSZ-5 to PPARγ LBD in 293T cells. **(D)** Luciferase reporter assay evaluating the transcriptional activity of PPARγ with TWSZ-5 and RSG treatment in 293T cells. **(E)** Luciferase reporter assay evaluating the transcriptional activity of wild type (WT) and site mutated PPARγ with or without TWSZ-5 treatment in 293T cells.

MD simulation (Figures 4A,B) demonstrated that TWSZ-5 stabilizes a distinct conformational state within the PPARγ LBD. Root-mean-square deviation (RMSD) analysis revealed that the PPARγ-ligand complex achieved equilibrium after 10 ns of simulation, with subsequent fluctuations stabilized within 1.8 Å (Figure 4A). This minimal deviation indicates robust structural integrity and stable ligand binding throughout the 100 ns trajectory. Hydrogen bond occupancy analysis highlighted the pivotal role of intermolecular interactions in stabilizing the ligand-protein complex. The number of hydrogen bonds between the ligand and PPARγ ranged from 0 to 6, with an average occupancy of approximately three bonds throughout the simulation (Figure 4B). This sustained hydrogen-bonding network underscores the ligand’s ability to engage key residues in the novel binding pocket. Further evaluation of global conformational dynamics via radius of gyration (Rg) and solvent-accessible surface area (SASA) demonstrated minor fluctuations (Supplementary Figures S4A,B), suggesting localized structural rearrangements while maintaining overall compactness. These results demonstrate that the PPARγ-TWSZ-5 complex adopts a stable conformation characterized by sustained hydrogen bonding, limited global flexibility, and localized rigidity in key binding regions. This dynamic profile supports the ligand’s role as a partial agonist engaging the novel allosteric pocket with high structural fidelity.

Subsequently, microscale thermophoresis (MST) was employed to assess the binding affinity of TWSZ-5 to the LBD of PPARγ in 293T cells. The findings demonstrated that TWSZ-5 could bind to the LBD of PPARγ with a Kd value of 4.87 μM in mammalian cells (Figure 4C). This observation signifies that TWSZ-5 can directly bind to PPARγ. In the luciferase reporter system, TWSZ-5 was found to exhibit dose-dependent transcriptional activity (Figure 4D). At a concentration of 100 nM, TWSZ-5 achieved about 15% of the activity observed for RSG at the same concentration. This reduced efficacy, coupled with the absence of full helicoidal repositioning, classifies TWSZ-5 as a partial PPARγ agonist. To further verify the binding site specificity, a luciferase reporter assay was conducted with alanine point mutant PPARγ proteins (Ser342A, Gln345A, Lys262A, Lys261A, and Ile262A). As shown in Figure 4E, the Ser342A, Gln345A, Lys263A, and Lys261A mutant completely blocked the TWSZ-5-induced transcriptional activation of PPARγ, suggesting that the Ser342, Gln345, Lys263, and Lys261 residues are the critical sites for TWSZ-5 binding to PPARγ. These findings align computationally derived binding stability with functional validation, underscoring the compound’s unique activation profile.

### 3.5 TWSZ-5 promoted beige adipocyte differentiation *in vitro*

**FIGURE 5:**
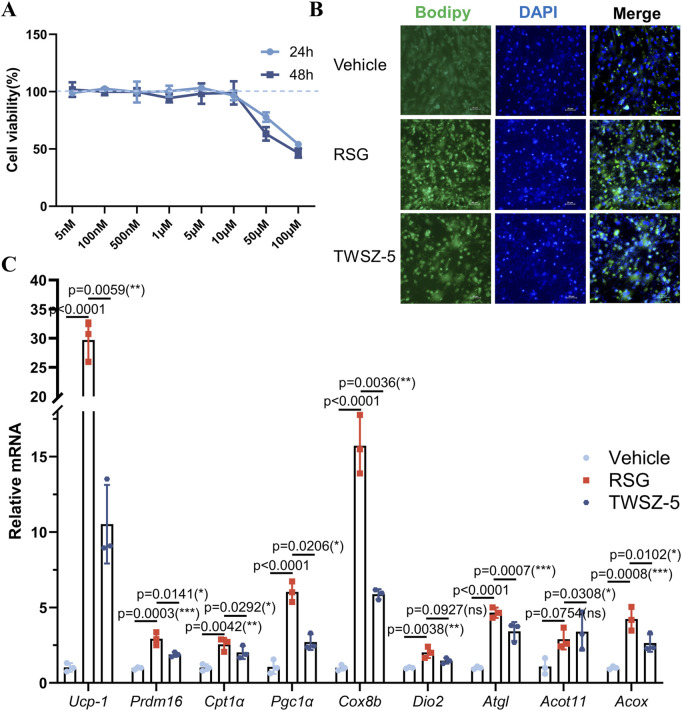
TWSZ-5 effectively promoted the beige adipocyte differentiation *in vitro*. **(A)** The cytotoxicity of TWSZ-5 on ADSCs. **(B)** BODIPY staining of the content of lipid droplets. **(C)** Beige cell-related marker gene expressions were evaluated by qPCR.

Biological evaluation of TWSZ-5 revealed its favorable safety and pro-adipogenic activity. Cytotoxicity assays demonstrated negligible effects on adipose-derived stem cells (ADSCs) at concentrations ≤10 μM over 24 and 48 h, confirming its biocompatibility (Figure 5A). Bodipy fluorescence staining of adipogenically induced ADSCs showed a marked increase in lipid droplet accumulation in TWSZ-5-treated groups compared to controls, indicating enhanced adipogenic differentiation (Figure 5B). Quantitative PCR further revealed a beige adipocyte-specific transcriptional profile, with significant upregulation of key markers including *Ucp1*, *Prdm16*, *Cpt1α*, *Pgc1α*, *Cox8b*, and *Acot11* (Figure 5C). Collectively, these data establish TWSZ-5 as a potent inducer of beige adipogenesis, highlighting its therapeutic potential for metabolic disorders such as obesity and diabetes.

## 4 Discussion

The discovery of a novel ligand-binding pocket in PPARγ LBD offers intriguing insights into the structural basis of partial agonists and provides a strategic framework for future virtual screening campaigns. Traditionally, the canonical orthosteric binding site of PPARγ, located between helices H3, H11, H12, and the β-sheet, has been the focus of agonist development (Shang and Kojetin, 2021; Miyachi, 2023). Full agonists like rosiglitazone, which occupy the orthosteric site, induce robust H12 stabilization and coactivator recruitment (Nolte et al., 1998; Lee et al., 2017). Helix 12 is highly flexible, and its stabilization with ligands induces co-activator recruitment and full activation of PPARγ. Therefore, this confirmation resulted in adipogenic side effects, including weight gain, edema, and osteoporosis (Bilandzic et al., 2016; Bansal et al., 2020; Kounatidis et al., 2025). Partial agonists, however, act on PPARγ through an H12-independent mechanism. For example, MRL-24 is positioned at a contact distance to Glu343, Ile341, Leu340, and Met348, and thus does not robustly stabilize H12, but stabilizes the β-sheet region of the LBD (Bruning et al., 2007). Saikosaponin A, a novel selective PPARγ agonist, has been shown to form direct hydrogen bonds with Arg280, Glu259, Ser342, Glu342, Glu291, and Glu295 on the helixes H3 and H2’ (Tian et al., 2024). This selective modulation has the potential to preserve the insulin-sensitizing effects while mitigating adverse outcomes. This hypothesis is supported by the correlation between ligand-binding poses in this pocket and transcriptional profiling results from analogous compounds (Li et al., 2023). Here, we used six widely used algorithms (Fpocket, DeepSite, CavityPlus, DoGSiteScorer, CASTpFold, and POCASA) for detecting the binding pocket of PPARγ LBD. The results demonstrated that the pocket 6–5 (H2-H3-β sheet region) was the most druggable, thereby signifying a substantial advancement in understanding the structural plasticity and pharmacological modulation of PPARγ.

The pocket 6-5 exhibits higher hydrophobicity and reduced polarity compared to the classical binding cavity, explaining the enrichment of halogenated aromatic moieties in our top-ranked virtual screening hits. This hydrophobicity-driven binding mode minimizes entropic penalties associated with ligand desolvation, a critical factor for optimizing partial agonist pharmacokinetics (Patil et al., 2010). This unique arrangement suggests a cooperative mechanism whereby ligand occupancy in the H3-H2-H2’ cavity promotes the repositioning of the AF-2 helix, a critical step for coactivator recruitment and transcriptional activation (Shang and Kojetin, 2021; Sönmezer et al., 2021). The pocket 6-5 exhibit demonstrated a reduction in coactivator binding in the simulations, which is consistent with experimental data indicating a decrease in transactivation of adipogenic genes (*Ucp1*, *Prdm16*, *Cpt1α*, *Pgc1α*, *Cox8b*, and *Acot11*). The observed structural features highlight the functional significance of this pocket in mediating action of PPARγ.

Natural products hold particular promise in the identification of PPARγ partial agonists due to their inherent structural diversity and evolutionary adaptation for bioactivity (Villarroel-Vicente et al., 2021; Ma et al., 2022). In contrast to synthetic libraries, natural compound repositories are enriched with chemically novel scaffolds that frequently exhibit polypharmacology, a feature that is critical for modulating multifaceted pathways such as adipose browning and metabolic regulation (Abel et al., 2002; Bauer et al., 2010; Grisoni et al., 2018). For instance, dihydromyricetin has been demonstrated to serve as a protective agent against weight gain induced by a high-fat diet (HFD) by modulating the PPARγ/SIRT1/PGC-1α pathway (Li et al., 2018; Leng et al., 2022). This effect is accompanied by an increase in the expression of browning-related genes and proteins in a model of obesity. Lycopene has been demonstrated the capacity to activate PPARγ while concurrently targeting PGC1α (Zhu et al., 2020a). This synergy has been shown to promote mitochondrial biogenesis and thermogenesis without inducing excessive adipogenesis. This multi-target engagement aligns with the partial agonist paradigm, as natural ligands may subtly rebalance PPARγ conformational dynamics, favoring coactivator recruitment profiles that enhance “healthy” adipose remodeling over lipid storage. Furthermore, natural products frequently demonstrate enhanced safety profiles in comparison to synthetic full agonists. This is substantiated by the diminished hepatotoxicity and cardiometabolic risks exhibited by herbal PPARγ modulators, such as resveratrol derivatives (Okla et al., 2017; Parsamanesh et al., 2021). Our virtual screening pipeline capitalized on these advantages by prioritizing natural compounds with intermediate binding affinities to the allosteric pocket. The findings indicated that the Ginsenoside Rg5 (TWZS-5) compound was successfully identified, and further analysis revealed its capacity to induce partial activation of the PPARγ and increase the expression of brown genes. This approach not only accelerates the identification of safer, tissue-selective PPARγ modulators but also bridges traditional pharmacopeias with modern structure-based drug design, offering a sustainable strategy to combat obesity-related metabolic disorders.

Prior studies have established that ginsenosides, such as Rg3, RK3, and Rb1, exhibit PPARγ-dependent antidiabetic and anticancer effects. For instance, ginsenoside Rg3 was effective in the inhibition of adipocyte differentiation, a process that was found to be associated with the suppression of PPAR-γ activity (Hwang et al., 2009). However, in another study reported that ginsenoside Rg3 binds directly to PPARγ, improving adiponectin signaling and ameliorating diabetic cardiomyopathy (DCM) in db/db mice (Zhang et al., 2023). This discrepancy underscores the disease-specific modulation of PPARγ signaling pathways by Rg3, suggesting that its therapeutic outcomes are contingent upon the pathophysiological environment and tissue-specific PPARγ interactions. Furthermore, ginsenoside Rb1 has been observed to induce a pro-neurogenic microglial phenotype in male mice exposed to chronic mild stress, with this effect being associated with the activation of PPARγ (Zhang et al., 2021). Ginsenoside Rk1 has been demonstrated to enhance endothelial function in diabetes by activating PPARγ (Miao et al., 2024). These studies suggest that the structure of ginsenosides may allow them to selectively influence pathways related to PPARγ. Our work builds upon these findings by identifying a novel binding pocket (pocket 6–5) in PPARγ and characterizing ginsenoside Rg5 as a partial agonist targeting this site. Unlike Rg3, which engages the canonical Y-shaped pocket (Zhang et al., 2023), Rg5 stabilizes a distinct conformation at the H3-H2’-β-sheet interface, inducing submaximal transcriptional activation while selectively upregulating thermogenic genes including Ucp1, Pgc1α, and Prdm16. This partial agonism avoids robust H12 stabilization, a hallmark of full agonists linked to adipogenic side effects, thereby reconciling the mechanistic gap left by earlier studies. These advances position ginsenoside Rg5 as a next-generation SPPARγM with translational potential for metabolic disorders, addressing the limitations of both classical TZDs and earlier ginsenoside candidates.

Ginsenoside Rg5 is a rare ginsenoside isolated from ginseng (*Panax ginseng* C.A. Meyer). The substance is characterized by its augmented membrane permeability and metabolic stability in comparison with glycosylated ginsenosides (Sugita et al., 2022). A growing body of research has demonstrated the significant pharmacological activities of ginsenoside Rg5, such as anti-inflammatory (Zhu et al., 2020b; Heo et al., 2023; Yu et al., 2023), anti-diabetic (Wei et al., 2020), and anti-cancer effects (Kim and Kim, 2015). Notably, ginsenoside Rg5 has shown a safe profile in some preclinical studies (Liu et al., 2021). Our findings extend this understanding by demonstrating that ginsenoside Rg5 preferentially engages the newly identified allosteric pocket, inducing a conformational shift that upregulates *Ucp1*-dependent thermogenesis in ADSC while attenuating PPARγ-driven adipogenic transcription including *Prdm16*, *Cpt1α*, and *Pgc1α*. Given its natural origin and multi-target engagement, ginsenoside Rg5 represents a promising lead compound for developing safer PPARγ modulators. It provides a phytochemical framework for engineering derivatives with optimized tissue specificity for obesity-related metabolic disorders. Future studies should validate ginsenoside Rg5 *in vivo*, particularly in models of diet-induced obesity, to assess its efficacy in ameliorating metabolic dysregulation while circumventing the side effects characteristic of classical PPARγ agonists. Collectively, our findings establish a structure-guided strategy to dissociate PPARγ′s metabolic benefits from adverse effects. This combinatorial strategy will facilitate the screening of PPARγ partial agonists.

## 5 Conclusion

This study identified a novel binding pocket (pocket 6–5) within the PPARγ ligand-binding domain through the integration of multiple computational algorithms. The pocket’s unique hydrophobic-polar residue architecture supports H12-independent partial agonism, bypassing classical adverse effects. Structure-based virtual screening of natural products revealed TWSZ-5 as a potent partial agonist that stabilizes this pocket via key hydrogen bonds (Ser342/Gln345), inducing a receptor conformation associated with dose-dependent partial activation (achieving one-third of the transcriptional activity of rosiglitazone). Functional validation demonstrated TWSZ-5’s ability to selectively upregulate beige adipogenesis markers (*Ucp1*, *Pgc1α*) without cytotoxicity at concentrations ≤10 μM. These findings establish pocket 6–5 as a therapeutic target and position TWSZ-5 as a lead compound for developing safer, tissue-selective PPARγ modulators, advancing precision drug design for metabolic disorders.

## Data Availability

The original contributions presented in the study are included in the article/Supplementary Material, further inquiries can be directed to the corresponding authors.
