# Supplementary figures and images for: Discovery of a novel binding pocket in PPARγ for partial agonists: structure-based virtual screening identifies ginsenoside Rg5 as a partial agonist promoting beige adipogenesis
Source: Front Chem. 2025 May 8;13:1579445. doi: 10.3389/fchem.2025.1579445 (PMC12095147; doi:10.3389/fchem.2025.1579445)

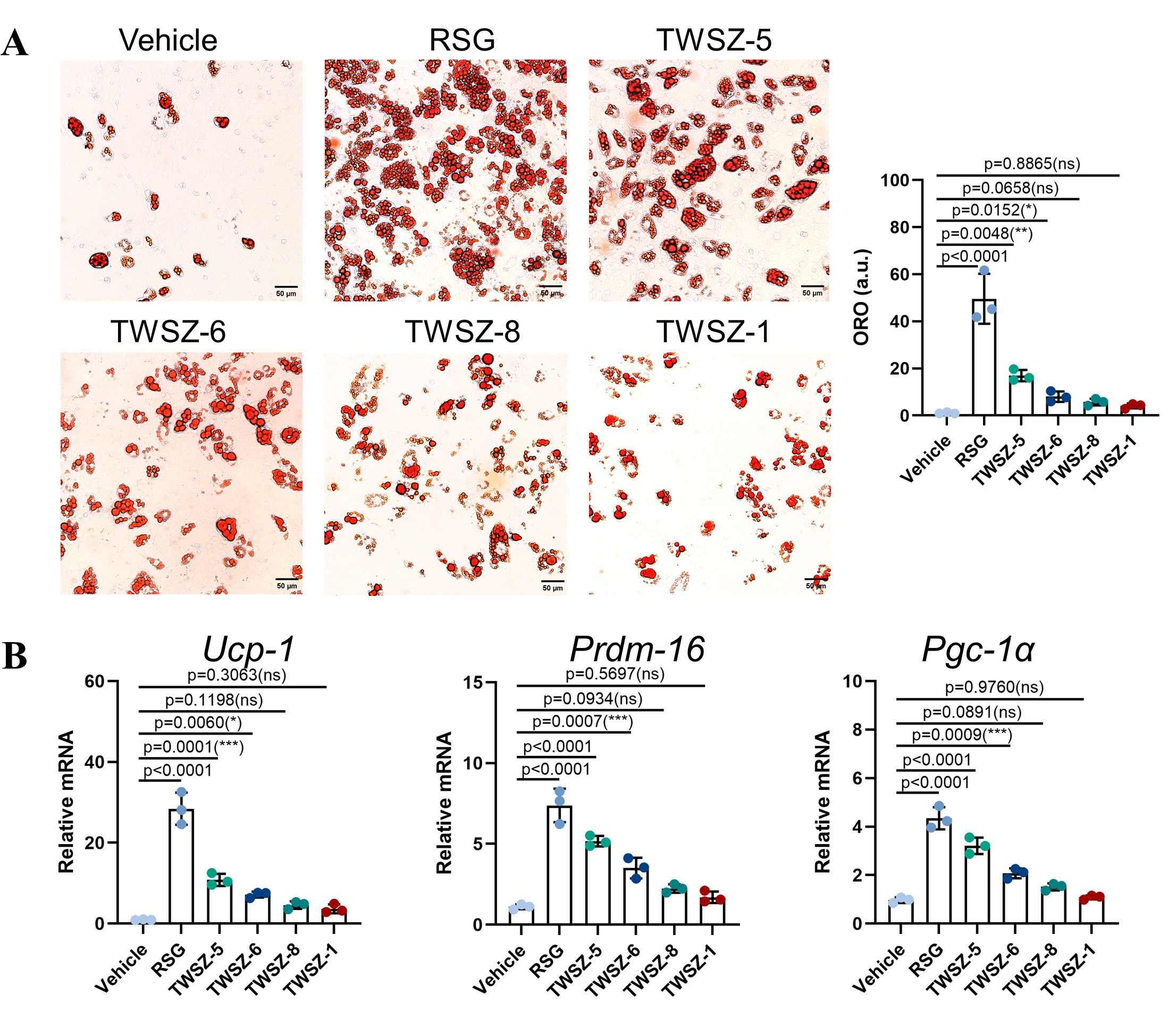

Supplement: Supplementary file 1 [file Image3.tif]

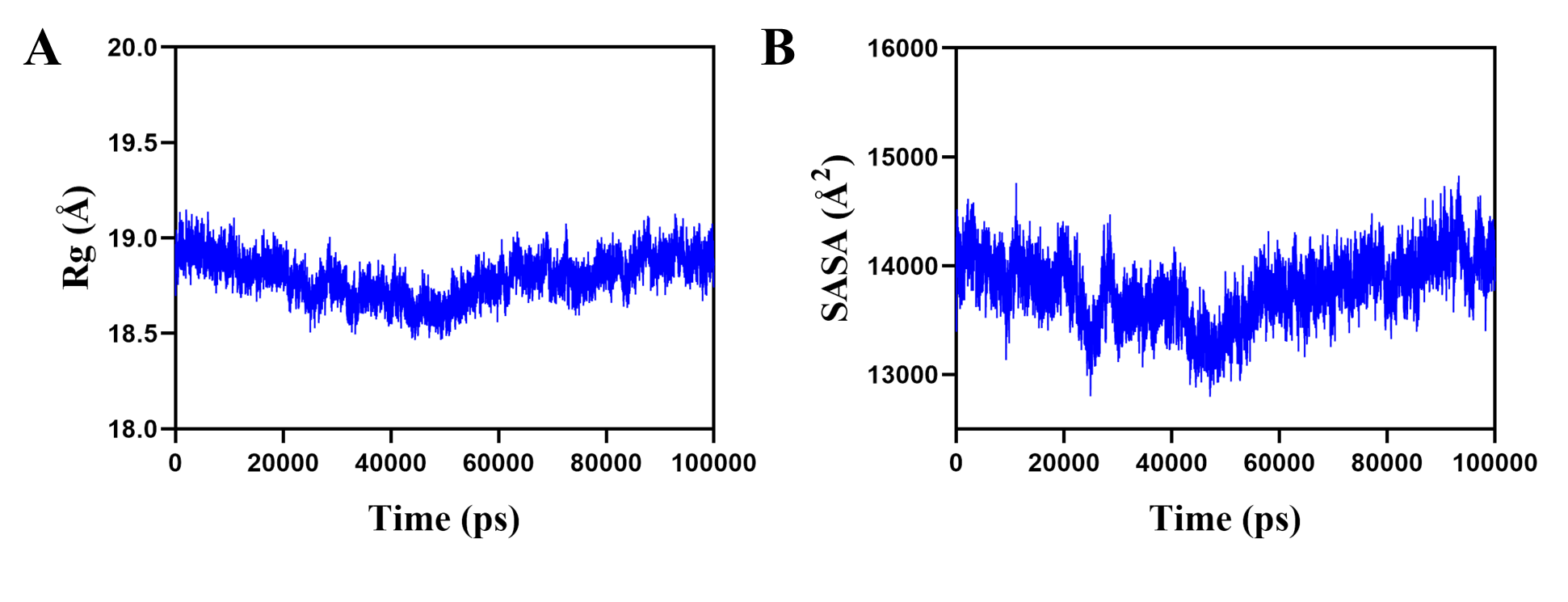

Supplement: Supplementary file 2 [file Image4.tif]

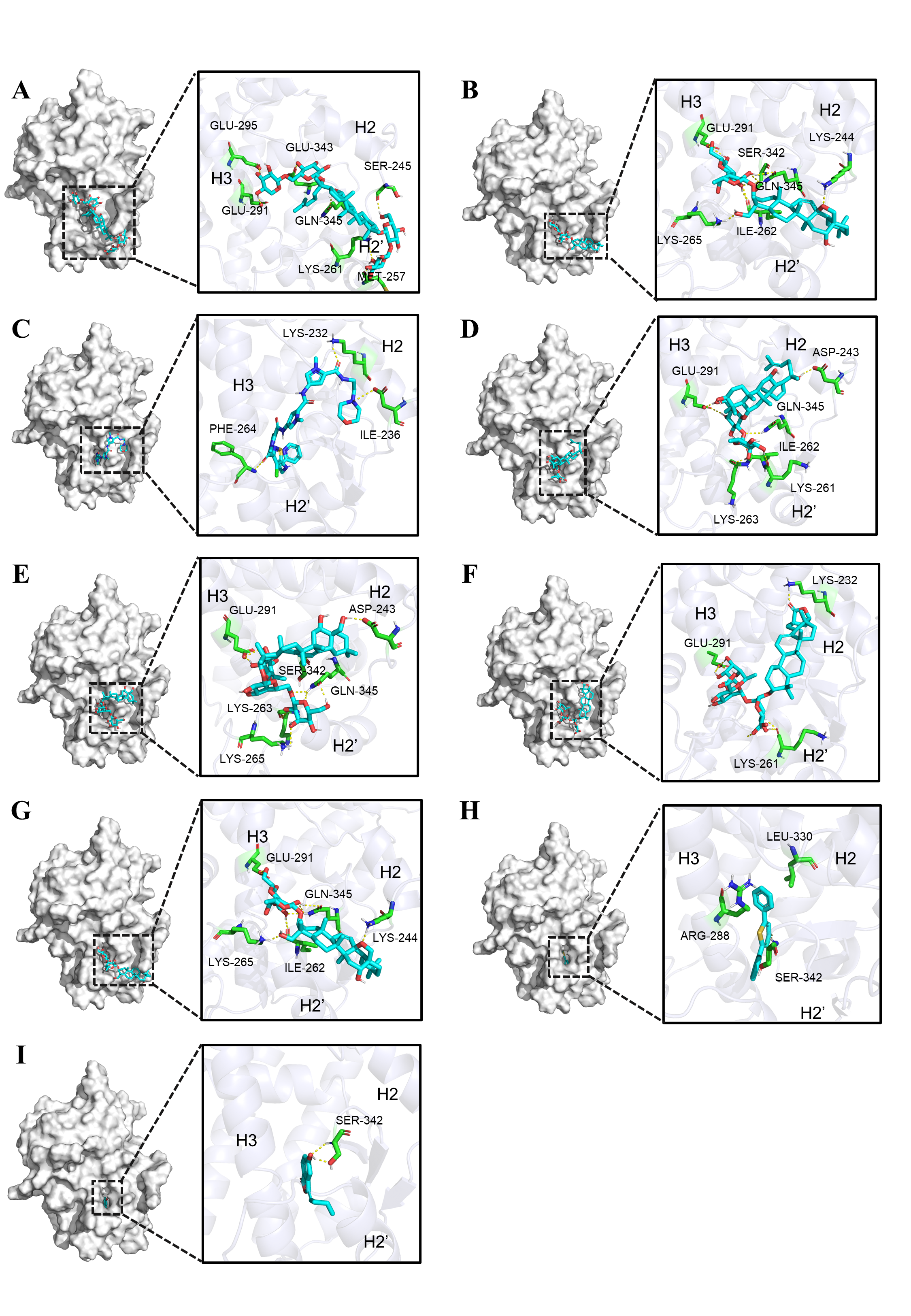

Supplement: Supplementary file 3 [file Image2.tif]

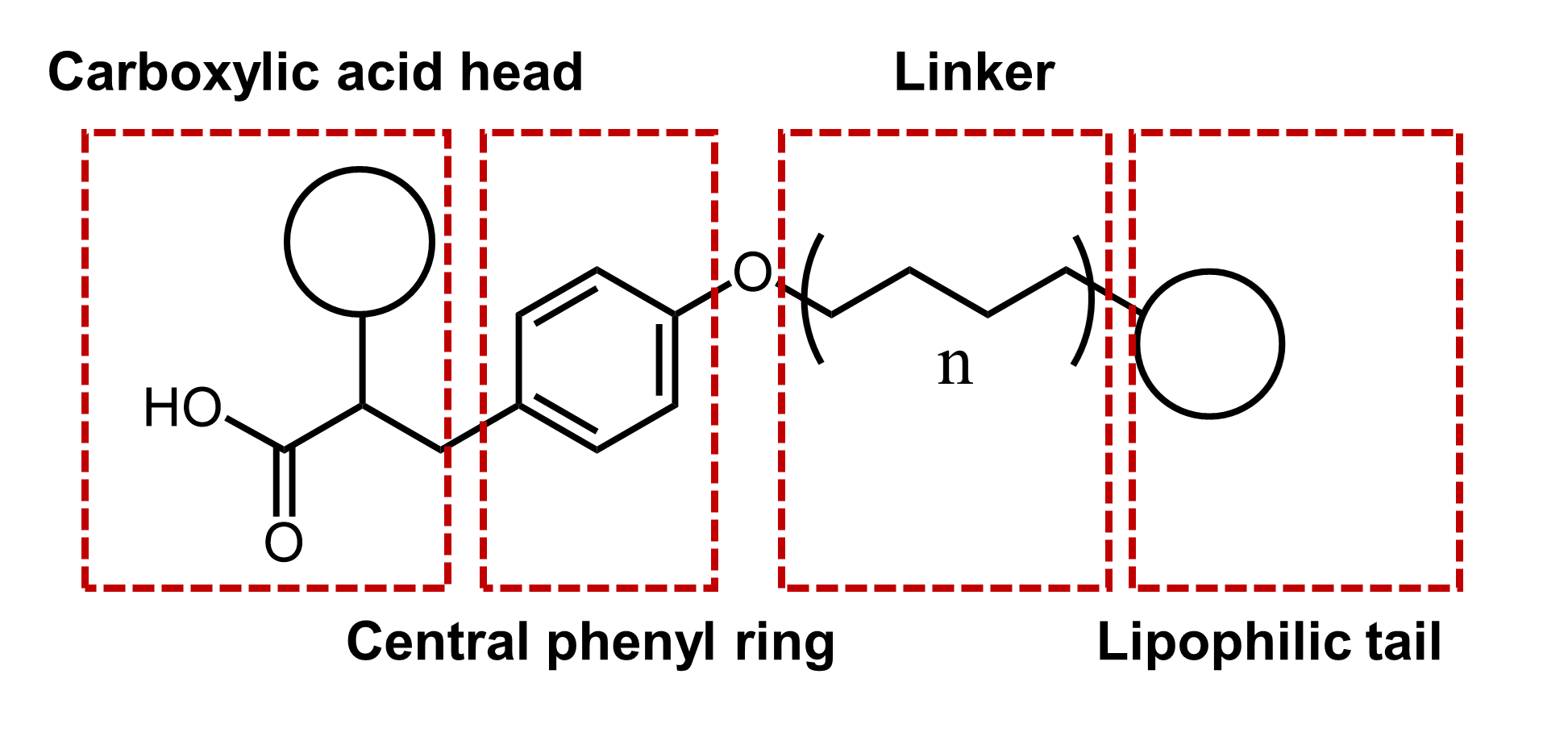

Supplement: Supplementary file 4 [file Image1.tif]
